# Implementation of a Teledermatology Electronic Consultation Program to Improve the Care of Patients with Inflammatory Bowel Disease

**DOI:** 10.1089/tmr.2023.0060

**Published:** 2024-01-24

**Authors:** Ana Echarri, Carmen Pradera, Gema Molina, Carlota Gonzalez Moure, Cristina De las Heras, Rebeca Fraga Iriso, Virginia Ollero, Cristina Echevarria, Javier Muñiz

**Affiliations:** ^1^ Department of Gastroenterology, Complejo Hospitalario Universitario de Ferrol, Ferrol, Spain.; ^2^ Department of Dermatology, Complejo Hospitalario Universitario de Ferrol, Ferrol, Spain.; ^3^ Departamento de Ciencias de la Salud e INIBIC, Universidad de A Coruña, CIBERCV, A Coruña, Spain.

**Keywords:** teledermatology, IBD care, e-health, e-consult

## Abstract

**Introduction:**

Skin lesions are a common extraintestinal manifestation associated with inflammatory bowel disease (IBD), although they may also appear as a complication of IBD treatment. Prompt referral to the dermatologist can be very helpful in practice. Teledermatology complements the traditional in-person health care modality, improving access to dermatological care.

**Objective:**

To evaluate the impact of a store-and-forward teledermatology electronic consultation (e-consult) program on the care of IBD patients.

**Methods:**

A retrospective study assessing the outcomes of our teledermatology program over its first 2 years of implementation.

**Results:**

A total of 39 consultations involving 33 patients (69.2% women, mean age 39.6 years [12–63]) were conducted. The mean number of teleconsultations was 2.8 per month in the initial implementation stage: 33 consultations were carried out in patients with Crohn's disease and 6 in ulcerative colitis. Only 18% of the patients had an active flare-up. The most frequent reason for the e-consult was paradoxical psoriasiform lesions (*n* = 13, 33.3%), commonly related with anti-tumor necrosis factor agents (70% of the patients) and hidradenitis suppurativa (*n* = 4, 10.3%). Resolution was achieved in 87% of patients, with a mean waiting time of 4.7 days (0–14). Almost all patients (97%) were satisfied with our program, and considered the referral through the program to be appropriate (92%). Best valued features were the reduced waiting time and the coordinated approach between the two departments involved.

**Conclusions:**

Dermatology e-consult is an efficient and useful means of optimizing IBD patient care.

## Introduction

Inflammatory bowel disease (IBD) is a chronic condition that mainly includes ulcerative colitis (UC) and Crohn's disease (CD), which share bowel inflammation and heterogeneous clinical manifestations at the intestinal and extraintestinal level. Extraintestinal manifestations (EIMs) often affect joints, skin, and eyes, having a significant impact on the patient's quality of life (QoL).

Cutaneous EIMs have been reported in 15–20% of patients with IBD. Although their incidence is similar in both UC and CD, there are some differences in the predominant manifestations, primarily that UC more frequently presents with pyoderma gangrenosum, whereas CD more often presents with erythema nodosum and aphthous stomatitis. In addition, adverse skin reactions, also called paradoxical responses, have been reported to develop in 5–10% of patients, most commonly after antitumor necrosis factor (anti-TNF) treatment.

Management of skin lesions should involve close collaboration with dermatologists to ensure prompt diagnosis and treatment, but long waiting times for dermatology appointments can lead to suboptimal management.^1^ Virtual care tools, such as teledermatology, can be an effective and easier way for early dermatologist collaboration.

According to a recent systematic review with meta-analysis, although teledermatology diagnoses are less reliable than those made in person, it can improve access, reduce costs, and triage patients to determine those requiring further in-person consultation.^2^ Moreover, in inflammatory skin diseases that are common in IBD patients, such as atopic dermatitis and psoriasis, telehealth is as effective as in-person care.^3^

Although previous studies have examined interdisciplinary teleconsultations between dermatological and general practice,^4,5^ such experiences are infrequent between specialized levels, and we found none establishing interdisciplinary teleconsultations between the gastroenterology and dermatology departments.

Accordingly, we developed a system that allows IBD specialists to arrange a rapid dermatological consultation regarding the treatment and management of skin lesions found in their patients (Supplementary Fig. S1).

The gastroenterologist takes some pictures of the lesion and enters the relevant clinical data into the patient's electronic health record (EHR), sending out an assessment request to the dermatology department. This generates an alert for the dermatologist, who assesses the received data and images and decides whether to directly issue an electronic prescription for treatment, or to make an appointment for an in-person consultation.

We hypothesized that this program may improve IBD patient care by facilitating easier dermatological access and avoiding emergency department visits, thus reducing costs for the health care system while enhancing patient satisfaction and QoL.

This study aimed to assess the results of the first 2 years after the implementation of a teledermatology program in our IBD unit.

## Methods

### Study design, sample size, and recruitment

A retrospective observational descriptive study was designed to assess the results of a teledermatology program between the IBD specialists and dermatologists at Ferrol University Hospital (Spain) in clinical practice, 2 years after its initial implementation (January 2019–2021).

After the launch of the program, all IBD patients with skin lesions were referred for dermatological evaluation through an online consultation. The dermatologist established the diagnosis and treatment based on the clinical data and images attached to the electronic consultation (e-consult) file and entered in the patient's EHR. All images were captured using a high-resolution compact digital mirrorless camera (Canon EOS M10-18 megapixel of resolution). We endeavored to ensure the protection of patient privacy during the image acquisition process.

The digital images consisted of three standard images (one medium-distance view, a close-up macro view, and a side view). The three images were obtained using autofocus without flash in a well-lit room (desk lamp could provide additional lighting). The background should be plain, light colored, and pattern free. All gastroenterologists in the IBD unit (*n* = 3) were trained in the process of image acquisition and uploading. Two dermatologists of the dermatology department of our hospital were in charge for reviewing and assessing the images, as well as solving the econsult.

The images were displayed on a full high-definition computer screen (1080p). Dermatologists could zoom and pan around the lesion. Color-scale adjustments were not allowed. Whenever the dermatologist considered it necessary, the patient was given an appointment for an in-person consultation.

During the first 2 years of the program, all patients who were referred to the dermatologist through the e-consult program were invited to fill out a satisfaction survey (provided in Supplementary Table S1) and were included in this study. Patients who did not have images of the skin lesion uploaded in their EHR were excluded.

Sample size was estimated for the number of consultations, not the number of patients, as the same patient may have had more than one consultation.

### Collected variables and statistical analysis

Collected variables included patient sociodemographic and clinical variables (age, gender, IBD diagnosis and type, previous surgeries, smoking habit, other EIM, current IBD treatment, type of cutaneous lesion and treatment, time to dermatologist appointment, and resolution of the lesion).

A descriptive analysis of the collected variables was carried out. Quantitative variables are expressed as mean and standard deviation or median and range (min–max or interquartile range). Qualitative variables are described as absolute value (*n*) and percentage.

### Ethical aspects

The study was carried out in accordance with the Declaration of Helsinki and applicable Spanish laws regulating biomedical research. The ethics committee of A Coruna/Ferrol evaluated and approved the study protocol.

## Results

The mean number of teledermatology consultations was 2.8 per month in the initial phase of implementation. A total of 39 consultations conducted in 33 patients were included in the study: two-thirds were women, most of the patients had CD, two-thirds were nonsmokers, and only a quarter of patients presented another EIM (rheumatological or ophthalmological). Most e-consults (*n* = 30) were related to patients presenting skin lesions for the first time (*n* = 77%). The characteristics of the patients are presented in Table 1.

**Table 1.** Baseline Sociodemographic and Clinical Characteristics of the Inflammatory Bowel Disease Patients Consulting a Dermatologist Through E-Consult

Characteristic	Value
Women (*n*, %)	27 (69.2)
Age, years (mean, SD)	39,59 (12.5)
IBD type (*n*, %)	
UC	6 (15.4)
CD	33 (84.6)
UC location (mode, IQR)	
Ulcerative proctitis (*n*, %)	2 (33.3)
Extensive UC, pancolitis (*n*, %)	4 (66.7)
CD location (mode, IQR)	
Ileal; L1 (*n*, %)	12 (36.4)
Colonic; L2 (*n*, %)	4 (12.1)
Ileocolonic; L3 (*n*, %)	15 (45.5)
Upper GI; L4 (*n*, %)	2 (6.1)
CD behavior (mode, IQR)	
Nonstructuring, nonpenetrating; B1	13 (39.4)
Stricturing; B2	18 (54.5)
Penetrating; B3	2 (6.1)
Perianal disease (*n*, %)	
Yes	13 (33.3)
No	26 (66.7)
Previous surgery (*n*, %)	
Yes	17 (43.6)
No	22 (56.4)
Smoker (*n*, %)	
Yes	14 (35.9)
No	25 (64.1)
Other EIMs (*n*, %)	
No	29 (74.4)
Rheumatological	8 (20.5)
Rheumatological + ophthalmological	2 (5.1)
Current treatment (*n*, %)	
Adalimumab	20 (51.3)
Infliximab	9 (23.1)
Infliximab + azathioprine	2 (5.1)
Azathioprine	6 (15.4)
No treatment	2 (5.1)

B1, disease behavior 1; B2, disease behavior 2; B3, disease behavior 3; CD, Crohn's disease; EIMs, extraintestinal manifestations; GI, gastrointestinal; IBD, inflammatory bowel disease; IQR, interquartile range; L1, localization 1; L2, localization 2; L3, localization 3; L4, localization 4; SD, standard deviation; UC, ulcerative colitis.

Almost one-fifth of the patients (18%) had an active flare-up. The most frequent reasons for teleconsultation were paradoxical psoriasiform lesions (*n* = 13, 33.3%) due to anti-TNF agents, hidradenitis suppurativa (*n* = 4, 10.3%), and atopic dermatitis (*n* = 4, 10.3%). Table 2 gives the diagnosis associated with each consultation.

**Table 2.** Diagnosis Associated with Each Consultation

Type of dermatological lesion	***n*** (%)
Psoriasiform reaction (*de novo*)	10 (25.6)
Hidradenitis	4 (10.3)
Atopic dermatitis	4 (10.3)
Psoriasis (exacerbation)	3 (7.7)
Acne/folliculitis	3 (7.7)
Lichen planus	3 (7.7)
Vasculitis	2 (5.1)
Seborrheic dermatitis	2 (5.1)
Eczema	2 (5.1)
Panniculitis	1 (7.7)
Rosacea	1 (7.7)
Drug-induced lupus	1 (7.7)
Melanocytic nevus	1 (7.7)
Vitiligo	1 (7.7)
Pyoderma gangrenosum	1 (7.7)
Total	39 (100)

Resolution was achieved in 87.2% of patients, and only one patient had to interrupt the anti-TNF therapy. The mean time to dermatologist response (either direct prescription or consultation) was 4.7 days (0–14); 16% of e-consults referred to the dermatologist required in-person evaluation, with a median waiting time of 7.2 days (5–16) compared with the 60 and 28 days on the current waiting list for a routine or priority appointment in the dermatology department of our hospital, respectively. The list of prescribed dermatological treatments is presented in Table 3.

**Table 3.** List of Prescribed Dermatological Treatments

Treatment	***n*** (%)
Topical treatment (corticosteroids)	31 (79.5)
Phototherapy	3 (7.7)
Methotrexate	3 (7.7)
Anti-TNF suspension	1 (2.6)
Ustekinumab	1 (2.6)
Surgery (nevus extirpation)	1 (2.6)
None	1 (2.6)

TNF, tumor necrosis factor.

Most of the patients (97%) rated this pathway positively, considering the e-consult referral very appropriate (92%; complete results are available in Supplementary Table S1). Patients highlighted the advantage of swiftness in both the resolution of the consultations and interaction between the two departments.

## Discussion

During the COVID-19 pandemic, telemedicine emerged worldwide as an indispensable resource to guarantee continuity of care of patients with multiple chronic diseases.^6^ As such, we should not relegate telehealth to a new location of care; rather, we need to explore its possibilities and find conditions wherein virtual care may help us in delivering better care to our patients.

Recent literature reviews and meta-analyses show that telemedicine can play a role in IBD care, with no significant differences reported in disease activity, remission rate, patient satisfaction, depression, self-efficacy, generic QoL, or medication adherence outcomes between telemedicine and standard care.^7,8^ Indeed one study reported that the telemedicine group had improved QoL and decreased the rate of in-person visits.^7^

In an effort to improve care of our IBD patients, we launched different services involving telemedicine and e-health to improve access to patient care and education.^7,9,10^ We recently identified delayed access to dermatological consultations that interfere in the care of our IBD patients presenting with skin lesions. Thus, in collaboration with the dermatology department, we developed a teledermatology program, which aimed to shorten the time to resolution of the patient's skin condition.

Teledermatology is a relatively well-established discipline that complements traditional in-person health care models. It had primarily been a resource aimed mainly at rural populations or underserved communities with less convenient accessibility,^11^ with a great number of e-consults between general physicians and dermatologists. It is likely to progressively expand to novel patient-centered telehealth care models, although to date, no studies have reported the use of teledermatology programs in routine IBD management. Our experience shows that teledermatology improves the access of IBD patients to dermatological care, facilitating patient management and decision making, with high effectiveness and patient satisfaction.

Teledermatology has been developed to work in either a live interactive or a store-and-forward format (SAFT). We preferred the SAFT for the flexibility it provides in the coordination of care, and because it allows asynchrony between the patient consulting with the IBD specialist and the diagnosis/prescription by the dermatologist. Thus, the patient can easily access the IBD unit, and be quickly referred to dermatology.

One of the criticisms of teledermatology is that there is no strong evidence that telematic diagnosis is as good as in-person diagnosis. Furthermore, several factors can interfere with the e-consult, including technology failures, poor photographic technique, and missing patient history,^2^ thus limiting the validity of the estimated diagnostic precision. Although a recent review with meta-analysis determined that telediagnosis is less precise than in-person consultations, it also concluded that there are still valid reasons for using teleconsultations, primarily to shorten waiting and consultation times in the case of low-risk conditions, as well as a way to provide an effective triage tool for those patients who really require an in-person evaluation.^2^

In both cases, the patient will receive an appropriate diagnosis and therapeutic management. Moreover, in inflammatory skin diseases commonly found in IBD patients, such as atopic dermatitis or psoriasis, telehealth is as effective as in-person care.^3^

In addition, in the dermatology e-consult we have implemented, the dermatologist can either electronically prescribe treatment immediately, when there is low risk of incorrect diagnosis, or make an urgent appointment for the patient when the condition requires a face-to-face assessment, having already collected most of the information that is needed for diagnosis; this constitutes an appropriate and fast triage tool. In our case, ∼90% of the consultations were resolved rapidly and efficiently, whereas only 16% of cases received a priority appointment for an in-person consultation.

Teledermatology is associated with a good level of patient satisfaction. In a recent study, even though more than half of surveyed patients preferred in-person examinations, the authors reported that >70% of the surveyed patients indicated at least equal satisfaction compared with in-person visits.^12^ In our study, most patients (97%) evaluated this pathway positively, considering the e-consult very appropriate (*n* = 92%) and highlighting the advantage of swiftness in both resolution of the consultations and interaction between the two departments.

The main limitation of this study is the small number of consultations that could be included during the first 2 years of functioning, which could not draw any illness-specific conclusions. However, this first data description allows for positive assessment of the experience of teledermatology e-consultation in our IBD unit.

In conclusion, our teledermatology program achieved a significant reduction in referral waiting times for dermatology consultations in IBD patients presenting with skin conditions, providing faster and more efficient management, and improving the QoL and satisfaction of patients. Implementing teledermatology in IBD care may represent a promising innovation to improve management of IBD patients with dermatological complications in real-world practice.

## Supplementary Material

Supplemental data

Supplemental data

## Abbreviations Used

B1: disease behavior 1
B2: disease behavior 2
B3: disease behavior 3
CD: Crohn's disease
EHR: electronic health record
EIMs: extraintestinal manifestations
GI: gastrointestinal
IBD: inflammatory bowel disease
IQR: interquartile range
L1: localization 1
L2: localization 2
L3: localization 3
L4: localization 4
QoL: quality of life
SAFT: store-and-forward format
SD: standard deviation
TNF: tumor necrosis factor
UC: ulcerative colitis
