# Supplementary material for: Implementation of a Teledermatology Electronic Consultation Program to Improve the Care of Patients with Inflammatory Bowel Disease
Source: Telemed Rep. 2024 Jan 24;5(1):12–7. doi: 10.1089/tmr.2023.0060 (PMC10927239; doi:10.1089/tmr.2023.0060)

## Fig. S1: The eConsult process through the hospital application: A) The gastroenterologist enters relevant clinical data into the electronic patient record; B) The gastroenterologist uploads photos of the skin lesions; C) The dermatologist fills in either the treatment for the patient or issues an appointment for an in-person consultation.


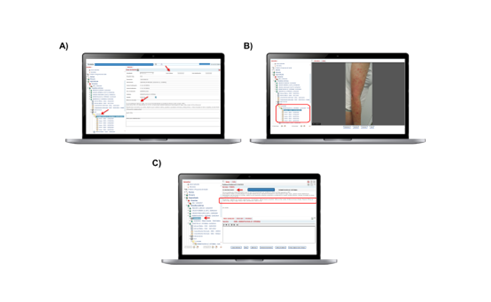

Supplement: Supplemental data [file Suppl_FigS1.docx]
