# Supplementary material for: Implementation of a Teledermatology Electronic Consultation Program to Improve the Care of Patients with Inflammatory Bowel Disease
Source: Telemed Rep. 2024 Jan 24;5(1):12–7. doi: 10.1089/tmr.2023.0060 (PMC10927239; doi:10.1089/tmr.2023.0060)
Supplement: Supplemental data [file Suppl_TableS1.docx]

Table S1. Summary of satisfaction survey results

| Question | n (%)  N=39 |
| --- | --- |
| Are you satisfied with the care you received for your skin problem?  YES  ……………………NO | 38 (97.4)  1 (2.6) |
| On a scale from 1 to 10 (1=bad; 10=excellent), please rate the usefulness of the eConsult to access dermatology care for your skin problem  Scored 10  Scored 9  Scored 8  Scored 7 | 24 (61.5)  12 (30.8)  2 (5.1)  1 (2.6) |
| Did you need to visit the Emergency Department for your skin problem?  YES  NO | 0 (0)   1. (100) |
| Which of the eConsult features do you appreciate the most?  Short waiting time  Coordinated care between the IBD unit and the dermatology department  Treatment effectiveness  Other | 25  10  0  0 |
